# A 12-Week, Multicenter, Randomized, Double-Blind, Placebo-Controlled Clinical Trial for Evaluation of the Efficacy and Safety of DKB114 on Reduction of Uric Acid in Serum

**DOI:** 10.3390/nu12123794

**Published:** 2020-12-10

**Authors:** Yu Hwa Park, Do Hoon Kim, Jung Suk Lee, Hyun Il Jeong, Kye Wan Lee, Tong Ho Kang

**Affiliations:** 1 Department of Oriental Medicine Biotechnology, Global Campus, Graduate School of Biotechnology and College of Life Sciences, Kyung Hee University, Gyeonggi 17104, Korea; best1rd@gmail.com (Y.H.P.); hoony3914@gmail.com (D.H.K.); 2 R&D Center, Dongkook Pharm. Co., Ltd., Gyeonggi 16229, Korea; ljs@dkpharm.co.kr (J.S.L.); jhi@dkpharm.co.kr (H.I.J.); lkw1@dkpharm.co.kr (K.W.L.)

**Keywords:** dietary supplements, asymptomatic hyperuricemia, DKB114, *Chrysanthemum indicum* Linn, *Cinnamomum cassia*, clinical trial, uric acid

## Abstract

This study sought to investigate the antihyperuricemia efficacy and safety of DKB114 (a mixture of *Chrysanthemum indicum* Linn flower extract and *Cinnamomum cassia* extract) to evaluate its potential as a dietary supplement ingredient. This clinical trial was a randomized, 12-week, double-blind, placebo-controlled study. A total of 80 subjects (40 subjects with an intake of DKB114 and 40 subjects with that of placebo) who had asymptomatic hyperuricemia (7.0–9.0 mg/dL with serum uric acid) was randomly assigned. No significant difference between the DKB114 and placebo groups was observed in the amount of uric acid in serum after six weeks of intake. However, after 12 weeks of intake, the uric acid level in serum of subjects in the DKB114 group decreased by 0.58 ± 0.86 mg/dL and was 7.37 ± 0.92 mg/dL, whereas that in the placebo group decreased by 0.02 ± 0.93 mg/dL and was 7.67 ± 0.89 mg/dL, a significant difference (*p* = 0.0229). In the analysis of C-reactive protein (CRP) change, after 12 weeks of administration, the DKB114 group showed an increase of 0.05 ± 0.27 mg/dL (*p* = 0.3187), while the placebo group showed an increase of 0.10 ± 0.21 mg/dL (*p* = 0.0324), a statistically significant difference (*p* = 0.0443). In the analysis of amount of change in apoprotein B, after 12 weeks of administration, the DKB114 group decreased by 4.75 ± 16.69 mg/dL (*p* = 0.1175), and the placebo group increased by 3.13 ± 12.64 mg/dL (*p* = 0.2187), a statistically significant difference between the administration groups (*p* = 0.0189). In the clinical pathology test, vital signs and weight measurement, and electrocardiogram test conducted for safety evaluation, no clinically significant difference was found between the ingestion groups, confirming the safety of DKB114. Therefore, it may have potential as a treatment for hyperuricemia and gout. We suggest that DKB114 as a beneficial and safe food ingredient for individuals with high serum uric acid. Trial registration (CRIS.NIH. go. Kr): KCT0002840.

## 1. Introduction

Hyperuricemia is a condition characterized by an abnormally elevated level of serum uric acid [1,2]. Uric acid is the final oxidation product of purine metabolism in humans in the absence of the hepatic enzyme uricase. Increased production and/or decreased uric acid excretion elevate serum uric acid level. The former is caused by an excessively purine-rich diet and purine metabolism overactivation, whereas the latter is caused by renal impairment and certain drugs. Hyperuricemia is diagnosed when serum uric acid level exceeds the limit of solubility (7.0 mg/dL) and increases the risk of monosodium urate or uric acid crystal deposition, which could result in acute gouty arthritis, gouty arthropathy, chronic tophaceous gout, uric acid urolithiasis, or gouty nephropathy [3,4,5,6].

Thus, long-term reduction of serum urate concentration to subsaturation level is vital in the treatment of gout [7]. Many epidemiological studies have shown that hyperuricemia and gout are associated with hypertension, cardiovascular disease, chronic kidney disease and diabetes, potentially through crystal-independent modes of action [8,9,10,11]. Clinical management of serum uric acid level often includes using a xanthine oxidase inhibitor (allopurinol) and uricosurics (probenecid and benzbromarone), which facilitate urinary excretion. However, their use can induce several adverse reactions, such as fever, skin rash, worsened renal function, and Stevens–Johnson syndrome [7,12,13,14,15,16,17,18].

To prevent gouty arthritis, cardiovascular disease, and renal failure, the Japanese guidelines for the management of hyperuricemia and gout recommend initiating pharmacologic urate-lowering therapy for asymptomatic hyperuricemia when serum urate level increases > 8.0 mg/dL [5]. The risk-benefit balance for using such drugs among gout-free patients with hyperuricemia is not favorable according to the guidelines for the management of hyperuricemia and gout [5,19,20,21]. Therefore, non-medication treatments, including a low-purine diet, exercise therapy, and natural products, are recommended for gout-free individuals with non-significantly high serum uric acid. In particular, it is commonly assumed that regular ingestion of dietary supplements is easier than dietetics or exercise therapy for individuals with non-significantly high serum uric acid and no gout pain. Although the majority of physicians have not regarded dietary supplements to be efficacious on hyperuricemia, the ingredients have been investigated for hypouricemic activity.

Our previous study demonstrated that DKB114 (a mixture of *Chrysanthemum indicum* Linn flower extract and *Cinnamomum cassia* extract) reduced serum uric acid level in normal rats and rats with PO-induced hyperuricemia and promoted excretion of uric acid in the urine, indicating that DKB114 has an anti hyperuricemic effect and may be a potent uricosuric agent. In addition, DKB114 inhibited xanthine oxidase activity and hepatic uric acid production in vitro and in vivo, as well as cellular uptake of uric acid in vitro [22].

Therefore, the present study evaluated the efficacy and safety of DKB114 intake in asymptomatic hyperuricemia (uric acid > 7.0 mg/dL) on blood uric acid reduction compared to Placebo (control foods) [23].

## 2. Materials and Methods

### 2.1. DKB114 Preparation

DKB114 (Dongkook Pharm. Co. Ltd, Suwon, Korea, Lot number is BPD170926-1) was prepared as described previously. Briefly, DKB114 was selected and prepared by mixing previously prepared *C. indicum* flower and *C. cassia* bark extracts at a weight ratio of 1:2 based on our previous report [22].

### 2.2. Study Design

This was a randomized, placebo-controlled, double-blinded clinical trial with a 3-month follow-up. The current protocol was registered at Neonutra Co. Ltd., Jongno, Korea (protocol number: DKP_DKB114, Version 3.3) on 14 February 2018, sponsored by Dongkook Pharm Co., Ltd. The participants were recruited from the Korean Medicine Hospital of Daejeon University, H Plus Yangji Hospital, and Bundang Jesaeng General Hospital. The study was fully conducted in accordance with the Declaration of Helsinki, and its protocol was approved by the Ethical Committee of the Institute Review Board of Daejeon University Dunsan Korean Medicine Hospital (Project identification code: DKP_DKB114; Submitted approval number: DJDSKH-17-BM-28; CRIS. NIH. go. Kr ID: KCT0002840).

Treatment was administered for 12 weeks, during which the participants were provided necessary guidance on maintaining daily diets and activities. During the clinical trial period, subjects were educated to maintain their usual diet, physical activity, and dietary intake. During the clinical trial period, subjects were educated to maintain their usual diet, physical activity, and dietary intake.

During the clinical trial testing period, the subjects were instructed to maintain the form of meals, physical activity, dietary intake was consumed as usual.

**Figure 1:**
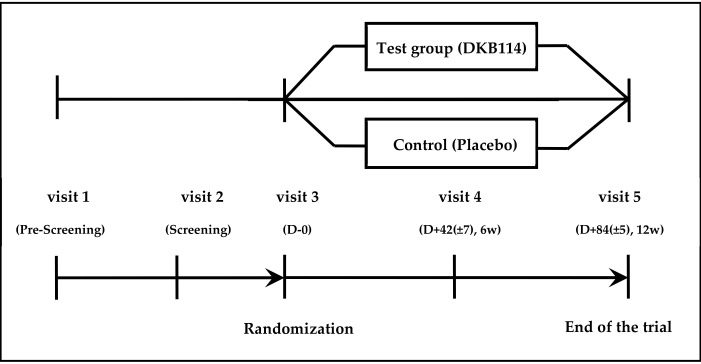
Flow diagram of the trial.

Foods related to *Cinnamomum cassia* and *Chrysanthemum indicum* Linn flower and health functional foods were not eaten. In addition, dietary survey papers were distributed to the test subjects at visit 3 and were made to fill out during the test period. The subjects were required to visit the hospital five times throughout the study. The assessments were scheduled as screening (week −2 to 0), baseline (week 0), interim (at the end of week 6), and final (at the end of week 12). The flow chart of the trial is shown in Figure 1.

### 2.3. Ethics Approval and Consent of the Participants

The trial protocol (DKP_DKB114, Version 3.3, dated 22 February 2019) was approved by the ethics committee of the Korean Medicine Hospital of Daejeon University, H Plus Yangji Hospital, Bundang Jesaeng general Hospital. Before the start of the clinical trial, the participant received information on the nature, scope, and expected results of the trial and provided written consent to participate. All clinical trial subject names were kept confidential and were recorded and confirmed with the number assigned during the trial. Subjects were informed that all test data were stored on a computer and treated strictly confidentially.

### 2.4. Participants

#### 2.4.1. Inclusion Criteria

Subjects who meet all the below criteria were qualified and enrolled in the study:

· Male or female adults aged between 20 and 75 years;
· Uric acid in serum concentration: more than 7.0 mg/dL to less than 9.0 mg/dL;
· Subjects who agree to participate in the clinical trial voluntarily and sign the informed consent form.

#### 2.4.2. Exclusion Criteria

Subjects who have one or more of the following characteristics were not considered for the study.

· Diagnosed with gout;
· Severe cerebrovascular disease or severe heart disease;
· Mental diseases such as schizophrenia, depressive disorder, and drug dependence;
· Alcohol abuse or dependence;
· Use of uric acid inhibitor (allopurinol, febuxostat, probenecid, etc.);
· Use of thiazide diuretics 4 weeks before the start of the test;
· History of urolithiasis;
· Hypertension not controlled by drugs, with greater than 160 mmHg systolic blood pressure or 100 mmHg diastolic blood pressure;
· Uncontrolled diabetes (HbA1c greater than 8%);
· Aspartate aminotransferase (GOT) or alanine aminotransferase (GPT) level 2 times higher than the normal upper limit;
· Creatinine higher than 2 mg/dL;
· Pregnant, lactating, or planning to become pregnant within 3 months;
· Participated in other clinical trials within 2 months of the start of the clinical trial or plan to participate in other clinical trials during this trial period;
· Difficulties participating in the trial as judged by the investigator.

#### 2.4.3. Withdrawal and Dropout

Cases in which the clinical trial was discontinued for a test subject in progress were as follows:

· Violation of inclusion criteria/exclusion criteria;
· Serious adverse event;
· Withdrawal of consent to participate;
· Use of drugs that may affect the results during the clinical period;
· Any safety reason;
· Pregnancy.

### 2.5. Recruitment

The participants will be recruited from the Korean Medicine Hospital of Daejeon University, H Plus Yangji Hospital, and Bundang Jesaeng General Hospital, South Korea. Recruitment for the clinical trial was advertised through posters in public places like hospitals and subways after approval from the institutional review board.

### 2.6. Randomization and Blinding

A randomization list was created by an independent analyst using block randomization in SAS version 9.2 (USA). Participants were given a random code and received treatment products marked with the same code. For balanced randomization between intake groups, the subjects of each group were equalized at a 1:1 ratio.

All participants and the research personnel were blinded to the assigned treatment until the end of the trial. In this clinical trial, no unblinding trials occurred.

### 2.7. Intervention

The test product was a 1 g purple tablet containing DKB114 functional ingredient (500 mg), crystalline cellulose, magnesium stearate, silicon dioxide, hydroxypropyl methylcellulose, glycerin fatty acid ester, titanium dioxide, lac color, and gardenia blue SB. A 1 g placebo product contained crystalline cellulose, silicon dioxide, magnesium stearate, hydroxypropyl methylcellulose, glycerin fatty acid ester, titanium dioxide, lac color, and gardenia blue SB. Participants were instructed to take 4 tablets each day, 2 in the morning and 2 at night, for a total dose of 4 g per day for 12 weeks (2 g/day as DKB114 functional ingredient). DKB114 or placebo was recommended to be administered after meals. In a rat model of calcium oxonate-induced hyperuricemia, the serum uric acid concentration decreased in the dkb114_200 mg/kg group. In addition, in the acute and chronic hyperuricemia animal models, it was confirmed that the serum uric acid concentration decreased, and the uric acid excretion concentration increased in the dkb114_200 mg/kg group. The value converted using the HED (human equivalent dose) conversion factor is 200/6.2 = 32.3 mg/kg, and when this value is calculated based on a 60 kg male, a dose of about 2.0 g is calculated. The value converted using the HED (human equivalent dose) conversion factor is 200/6.2 = 32.3 mg/kg, and when this value is calculated based on a 60 kg male, a dose of about 2.0 g is calculated.

HED (human equivalent dose) value was converted by using the conversion factor is to be 200/6.2 = 32.3 mg/kg, when calculating the value to 60 kg man is calculated based on a dose of about 2.0 g.

Based on these results, the daily intake in humans was determined as 2 g.

The DKB114 and placebo products were manufactured by COSMAX BIO Co., Ltd. (Gyeonggi, Korea) in a GMP-certified facility. The trial schedule is presented in Table 1.

### 2.8. Prohibited Concomitant Drugs and Therapies

The use of the following drugs and treatments could interfere with the evaluation of the safety, efficacy, and tolerability of the test product and were limited during the clinical trial.

· Uric acid inhibitors (allopurinol, febuxostat, probenecid, etc.);
· Uric acid release accelerator (urinon, narcaricin, etc.);
· Urate oxidase;
· Thiazide diuretics.

### 2.9. Outcome Evaluation

The following outcomes were evaluated by trained evaluators at each visit.

#### 2.9.1. Primary Outcomes

The mean uric acid in serum was compared before ingestion (visit 1: week-3) and after ingestion (visits 4, 5: weeks 6, 12). The degree of improvement in the DKB114 group and the placebo group was analyzed and compared to evaluate whether there was a statistically significant difference.

#### 2.9.2. Secondary Outcomes

The proportion of subjects with a uric acid serum concentration less than 7.0 mg/dL before ingestion (visit 1: week-3) and after ingestion (visits 4, 5: weeks 6, 12) was compared. Before ingestion (visit 2: week-2) and after ingestion (visits 4, 5: weeks 6, 12), the average xanthine oxidase activity in blood, blood sugar, C-reactive protein (CRP), homocysteine, TNF-α, IL-6, NO, and apoprotein B levels were compared. The degree of improvement in the DKB114 group and the placebo group was analyzed and compared to evaluate whether there was a statistically significant difference.

#### 2.9.3. Safety Outcomes

The safety outcome variables were adverse events, vital signs (blood pressure, pulse rate, body weight, etc.), clinical test results, and electrocardiogram results.

### 2.10. Sample Size

Among the existing studies conducted with a design similar to this clinical trial, the number of clinical subjects was calculated using the results of Hideki et al., a clinical trial using blood uric acid, which had the same endpoint as this clinical trial [24]. As a result, the minimum number of subjects was 35 per group. Considering a dropout rate of 12%, the number of clinical trial subjects to be enrolled per group for efficacy evaluation was 40, and the total number was 80.

### 2.11. Statistical Analysis

Statistical analyses were performed using SAS (version 9.4, SAS Institute, Cary, NC, USA).

The efficacy evaluation analysis and safety evaluation analysis were performed at a significance level of 0.05 with a two-sided test. If the *p*-value was <0.05, it was judged to be significant.

Uric acid in the serum—the primary efficacy evaluation—was analyzed using a paired *t*-test. The degree of change between groups at each visit was evaluated for statistically significant differences by conducting ANCOVA and two-sample *t*-tests.

The degree of change in blood xanthine oxidase activity, blood sugar, CRP, homocysteine, TNF-a, IL-6, NO, and apoprotein B level before and after ingestion were analyzed as the secondary efficacy evaluation using paired *t*-test. The degree of change between groups at each visit was evaluated for statistically significant differences by conducting ANCOVA and two-sample *t*-tests. The proportion of subjects with a uric acid concentration in the serum <7.0 mg/dL was compared and analyzed using the chi-squared test or Fisher’s exact test.

## 3. Results

This study was conducted at Daejeon University Dunsan Oriental Medicine Hospital, H Plus Yangji Hospital, and Bundang Jesaeng Hospital from February 2018 to July 2019. In this clinical trial, a screening evaluation was conducted on a total of 140 subjects to select suitable participants. A total of 80 subjects (40 subjects in the DKB114 group and 40 subjects in the placebo group) was randomly assigned to 60 screening eliminations. After 8 subjects withdrew consent, 4 in the test group and 4 in the control group, a total of 72 subjects completed the clinical trial (DKB114 group 36 subjects, placebo group 36 subjects).

**Figure 2:**
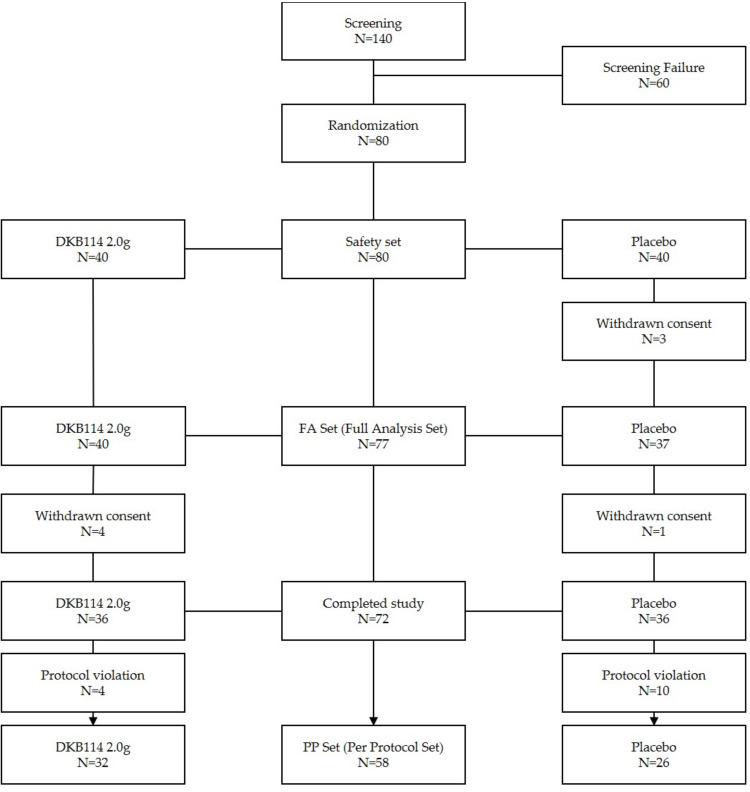
Disposition of clinical trial subjects.

In the per-protocol set (PP set), five people (four DKB114 groups, one placebo group) who dropped out after visit 4 were excluded. In addition, and 4 violations of more than 5 days of visit (4 placebo group) through the analysis group decision, 9 people with less than 80% compliance (3 DKB114 groups, 6 placebo group), and 1 overdose with greater than 130% secondary compliance (DKB114 group) were excluded. Thus, a total of 58 subjects (32 DKB114 groups, 26 placebo group) were included in the PP set (Figure 2)

All characteristics before ingestion, including demographic information of the subjects, were compared by ingestion group to identify factors with differences. Table 2 compares demographic information and pre-ingestion characteristics of clinical subjects.

The test group comprised 28 males (88.46%) and 4 females (12.50%), while the control group contained 23 males (88.46%) and 3 females (11.54%). There was no statistically significant difference between the intake groups. In terms of age, the DKB114 group averaged 41.84 ± 14.75 years, and the placebo group averaged 40.19 ± 12.53 years. There was no statistically significant difference between the intake groups (*p* = 0.6523). In addition, there were no statistically significant differences in smoking status, smoking amount, smoking period, and exercise status, demonstrating comparability between intake groups.

### 3.1. Clinical Parameters

#### 3.1.1. Primary Outcomes

Table 3 shows the results of the analysis of changes in serum uric acid measured at 0 weeks, 6 weeks, and 12 weeks after intake in the PP set. No significant differences between the DKB114 and placebo groups were observed in the amount of uric acid in serum after six weeks of intake. The mean 6-week uric acid in serum did not differ significantly between the DKB114 (7.47 ± 1.03 mg/dL) and placebo (7.54 ± 1.01 mg/dL) (*p* = 0.1734) groups. However, after 12 weeks of intake, the uric acid in serum of subjects in the DKB114 group decreased by 0.58 ± 0.86 mg/dL to 7.37 ± 0.92 mg/dL, whereas that in the placebo group decreased by 0.02 ± 0.93 mg/dL to 7.67 ± 0.89 mg/dL, a significant difference (*p* = 0.0229 *).

#### 3.1.2. Secondary Outcomes

The proportion of subjects with a uric acid serum concentration <7.0 mg/dL before ingestion (visit 1: week-3) and after ingestion (visits 4, 5: weeks 6, 12) was compared. Before ingestion (visit 2: week-2) and after ingestion (visits 4, 5: weeks 6, 12), the average xanthine oxidase activity in blood, blood sugar, CRP, homocysteine, TNF-α, IL-6, NO, and apoprotein B levels were compared. The degree of improvement between the DKB114 group and the placebo group was analyzed and compared to evaluate whether there was a statistically significant difference.

Table 4 shows the proportion of subjects with serum uric acid concentration <7.0 mg/dL at 0 weeks, 6 weeks, and 12 weeks of intake by PP set. Such patients numbered 12 (37.5%) in the DKB114 group and 5 (19.23%) in the placebo group after 6 weeks, and there was no statistically significant difference between groups. After 12 weeks, there were 11 such patients (34.38%) in the DKB114 group and 4 (15.38%) in the placebo group. There was no statistically significant difference between the intake groups.

However, the distribution of serum uric acid levels in recruited subjects (Table 5) showed that 12 weeks after intake, the DKB114 group tended to decrease serum uric acid levels compared to the placebo group.

Table 6 shows the results of the analysis of changes in blood xanthine oxidase activity, blood glucose, C-reactive protein (CRP), homocysteine, IL-6, NO, and apoprotein B measured at 0, 6, and 12 weeks of administration. In the analysis of CRP change, after 6 weeks of administration, the DKB114 group decreased by 0.01 ± 0.15 mg/dL (*p* = 0.7613), and the placebo group increased by 0.17 ± 0.73 mg/dL (*p* = 0.2402), with no statistically significant difference between the intake groups. After 12 weeks of administration, the DKB114 group increased by 0.05 ± 0.27 mg/dL (*p* = 0.3187), and the placebo group increased by 0.10 ± 0.21 mg/dL (*p* = 0.0324), a statistically significant difference (*p* = 0.0443 ^&^).

In the analysis of change in apoprotein B, after 6 weeks of administration, the DKB114 group decreased by 3.64 ± 19.55 mg/dL (*p* = 0.3007), and the placebo group decreased by 1.32 ± 9.64 mg/dL (*p* = 0.4917), with no statistically significant difference. After 12 weeks of administration, the DKB114 group decreased by 4.75 ± 16.69 mg/dL (*p* = 0.1175), and the placebo group increased by 3.13 ± 12.64 mg/dL (*p* = 0.2187), a statistically significant difference (*p* = 0.0189 ^$^).

In the analysis of changes in blood xanthine oxidase activity, after 6 weeks, the DKB114 group increased by 0.04 ± 0.66% (*p* = 0.7615), and the placebo group decreased by 0.13 ± 1.35% (*p* = 0.6233), with no statistically significant difference. After 12 weeks of administration, the DKB114 group increased by 0.04 ± 0.71% (*p* = 0.7768), and the placebo group decreased by 0.07 ± 1.02% (*p* = 0.7109), with no statistically significant difference.

In the analysis of blood glucose change, after 6 weeks of administration, the DKB114 group decreased by 2.41 ± 16.34 mg/dL (*p* = 0.4111), and the placebo group increased by 1.19 ± 13.41 mg/dL (*p* = 0.6542), with no statistically significant difference. After 12 weeks of administration, the DKB114 group decreased by 2.94 ± 15.32 mg/dL (*p* = 0.2863), and the placebo group decreased by 0.65 ± 11.10 mg/dL (*p* = 0.7664), with no statistically significant difference.

Regarding the amount of change in homocysteine, IL-6, and NO at each visit, there was no significant difference between the DKB114 group and the placebo group at 6 and 12 weeks of administration.

#### 3.1.3. Safety Evaluation

The safety evaluation was carried out as the main analysis of the safety set analysis, and a total of 80 subjects (40 subjects in the DKB114 group and 40 subjects in the placebo group) who consumed the food for the clinical trial at least once after being randomized was included in the analysis. In addition, clinical pathologic examination (serum chemistry test), vital signs (heart rate, blood pressure), and weight results were analyzed ( Table 7 and Table 8). No serious adverse reactions occurred, and there were no dropouts due to adverse reactions.

In the analysis of changes in total cholesterol during the serum chemistry test, after 12 weeks of intake, the DKB114 group decreased by 5.94 ± 17.00 mg/dL (*p* = 0.0432), and the placebo group increased by 10.03 ± 19.72 mg/dL (*p* = 0.0043), a significant difference (*p* = 0.0005). In the analysis of LDL cholesterol change, after 12 weeks of administration, the DKB114 group decreased by 8.89 ± 19.10 mg/dL (*p* = 0.0084), and the placebo group increased by 6.31 ± 21.07 mg/dL (*p* = 0.0812), a significant difference (*p* = 0.0020). All values were within the normal ranges, and there was no statistically significant difference between the administration groups in other serum chemical tests.

In the analysis of vital signs (pulse, blood pressure) and weight change, there were no statistically significant differences between intake groups at 6 weeks and 12 weeks.

## 4. Discussion

Hyperuricemia is diagnosed when serum uric acid level exceeds the limit of solubility (7.0 mg/dL), which increases the risk of monosodium urate or uric acid crystal deposition and can result in acute gouty arthritis, gouty arthropathy, chronic tophaceous gout, uric acid urolithiasis, or gouty nephropathy (6). In addition, hyperuricemia is a risk factor for cardiovascular diseases (25). Treatment of asymptomatic hyperuricemia is not necessary for most patients unless they have a very high level of uric acid or are otherwise at risk of complications, such as those with a personal or strong family history of gout, urolithiasis, or uric acid nephropathy. However, no direct role of hyperuricemia in the pathogenesis or outcome of these conditions was confirmed.

There is a need to develop a functional ingredient that can prevent gout and related complications caused by uric acid in the blood. In a previous study, we performed in vitro and in vivo tests using DKB-114, a natural functional ingredient. DKB114 markedly reduced serum uric acid level in normal rats and rats with PO-induced hyperuricemia while increasing renal uric acid excretion. Furthermore, it inhibited the activity of xanthine oxidase (XOD) in vitro and in the liver, in addition to reducing hepatic uric acid production. DKB114 decreased cellular uric acid uptake in oocytes, and HEK293 cells expressing human urate transporter (hURAT)1 and decreased the protein expression levels of urate transporters, URAT1, and glucose transporter, GLUT9, associated with reabsorption of uric acid in the kidney. DKB114 exerts antihyperuricemic effects and uricosuric effects, which are accompanied by a reduction in the production of uric acid and promotion of uric acid excretion via inhibition of xanthine oxidase activity and reabsorption of uric acid (22).

The present study aimed to investigate the antihyperuricemia efficacy and safety of DKB114 to evaluate its potential as a functional food ingredient. The present study performed a randomized controlled study and investigated the effect of oral administration of DKB114 on serum uric acid levels in 7.0–9.0 mg/dL with insignificantly high serum uric acid.

As an efficacy evaluation index, uric acid concentration in serum, the proportion of subjects with serum uric acid concentration <7.0 mg/dL, blood xanthine oxidase activity, blood glucose, CRP, homocysteine, TNF-α, IL-6, NO, and apoprotein B were measured. Among them, uric acid concentration in serum, CRP, and apoprotein B were clinically significantly improved in the DKB114 intake group compared to the placebo group. After 12 weeks of intake, the serum uric acid concentration in the DKB114 group was significantly decreased compared to before intake, but the placebo group had no statistical significance compared to before ingestion; as a result, a statistically significant difference was found between intake groups (*p* = 0.0229).

In the analysis of CRP change, after 12 weeks of intake, the DKB114 group increased by 0.05 ± 0.27 mg/dL (*p* = 0.3187), and the placebo group increased by 0.10 ± 0.21 mg/dL (*p* = 0.0324), a significant difference (*p* = 0.0443). C-reactive protein (CRP) is a protein that reacts with c-polysaccharide, a surface antigen of Streptococcus pneumoniae, and is one of the acute phase reactants whose concentration changes in a nonspecific response to inflammation or tissue damage. Through the intake of DKB114, CRP significantly changed compared to the placebo group, and DKB114 can be expected to act positively on the inflammatory response as well as uric acid reduction.

At 12 weeks after ingestion of apoprotein B, the level in the DKB114 group decreased, while that of the placebo group increased, showing a statistically significant difference between groups (*p* = 0.0189). Cardiovascular disease-related apoprotein B decreased through DKB114 intake but increased in the placebo group. There was no statistically significant difference between the groups in the proportion of subjects with serum uric acid concentration <7.0 mg/dL. However, after 12 weeks of ingestion, 11 patients in the DKB114 group and 4 patients in the placebo group showed a tendency to improve.

DKB114 showed no statistically significant difference in adverse reactions between groups, all of which were mild. Thus, it was judged that there was no effect of the food on the clinical results.

In the clinical pathology test, vital signs and weight measurement, and electrocardiogram test conducted for safety evaluation, no clinically significant difference was found between the ingestion groups, confirming the safety of DKB114. These findings indicate its potential as a treatment for hyperuricemia and gout. However, the group of subjects recruited (DKB114, controls) could be small to draw conclusions about the efficacy of DKB114. In the process of selecting only those who meet the per protocol (PP) set criteria among recruited subjects, the number of subjects in the controls group decreased. Based on the results of the current clinical trial, additional trials are being planned by increasing the number of subjects.

## 5. Conclusions

For the treatment of asymptomatic hyperuricemia in Japan, it is recommended to achieve blood uric acid concentration <7.0 mg/dL more and less than 9.0 mg/dL, and medicines are recommended for those with blood uric acid concentration ±9.0 mg/dL [5]. The United States and Europe do not specifically recommend drug treatment for asymptomatic hyperuricemia [19,20]. However, if the blood uric acid level is more than 9.0 mg/dL, the possibility of kidney damage increases, so uric acid-lowering medication is considered according to the patient [25]. Reducing uric acid production and increasing uric acid excretion may be a useful therapeutic approach for hyperuricemia treatment [26]. Currently, xanthine oxidase inhibitors such as allopurinol and febuxostat and uricosuric agents such as benzbromarone and probenecid are used for clinical hyperuricemia treatment [26]. However, these drugs are poorly tolerated and induce side effects, such as drug allergies, gastrointestinal symptoms, kidney disease, hypersensitivity syndrome, and hepatotoxicity [27,28,29,30]. Thus, new therapeutic strategies with minimal side effects are needed. An increasing interest in natural products, such as herbal medicines, has elucidated new approaches that can overcome these limitations, suggesting natural products as a promising pool of candidates for drugs and functional foods for hyperuricemia management [30].

We suggest DKB114 as a beneficial and safe food ingredient for individuals with high serum uric acid.

## Figures and Tables

**Table 1:** Trial schedule.

Period		Pre-Screening	Screening	Active Treatment		
Visit		1	2	3	4	5
Week		−3	−2	0	6	12
Window period					+/−7	+/−5
Written consent		✓				
Demographic survey			✓			
Medical history/drug administration		✓	✓	✓		
Physical examination			✓		✓	✓
Vital signs (pulse rate, blood pressure)		✓	✓	✓	✓	✓
ECG			✓			✓
Body measurement	Height			✓		
	Weight, BMI, waist circumference			✓	✓	✓
Drinking habits questionnaire				✓	✓	✓
Meal guidance and dietary survey				✓	✓	✓
Clinical pathology			✓			✓
Pregnancy test			✓			
Effectiveness evaluation	Uric acid in serum	✓			✓	✓
	Xanthine oxidase activity in serum		✓		✓	✓
	Blood sugar		✓		✓	✓
	CRP		✓		✓	✓
	Homocysteine		✓		✓	✓
	TNF-α		✓		✓	✓
	IL-6		✓		✓	✓
	NO		✓		✓	✓
	Apoprotein B		✓		✓	✓
Suitability of subjects		✓	✓	✓		
Randomization				✓		
Test and control food prescription				✓	✓	
Adverse reaction assessment					✓	✓
Compliance assessment					✓	✓
Concomitant medication and combination therapy confirmation					✓	✓

ECG: electrocardiogram; CRP: C-reactive protein; IL-6: interleukin-6; TNF-α: tumor necrosis factor alpha; NO: nitric oxide.

**Table 2:** Baseline characteristics (per-protocol set (PP) set).

		DKB114	Placebo	Total	*p*-Value
		N = 32	N = 26	N = 58	
Sex n (%)	Male	28 (87.50)	23 (88.46)	51 (87.93)	1.0000 ^‡^
	Female	4 (12.50)	3 (11.54)	7 (12.07)	
Age (years)	Mean ± SD	41.84 ± 14.75	40.19 ± 12.53	41.10 ± 13.71	0.6523 *
	Min, max	20.00, 69.00	23.00, 67.00	20.00, 69.00	
Smoking n (%)	Non-smoker	18 (56.25)	14 (53.85)	32 (55.17)	0.9367 ^†^
	Ex-smoker (not smoking for more than 6 months)	5 (15.63)	5 (19.23)	10 (17.24)	
	Smoker	9 (28.13)	7 (26.92)	16 (27.59)	
Smoking amount	Smoker, (___) cigarettes/1 day				
	Mean ± SD	9.89 ± 7.72	10.14 ± 5.81	10.00 ± 6.73	0.9434 *
	Min, max	1.00, 20.00	3.00, 20.00	1.00, 20.00	
Smoking period	Smoker, (___) year				
	Mean ± SD	12.56 ± 6.84	11.43 ± 10.42	12.06 ± 8.29	0.7977 *
	Min, max	2.00, 21.00	2.00, 30.00	2.00, 30.00	
Exercise or not n (%)	No	12 (37.50)	10 (38.46)	22 (37.93)	0.8633 ^‡^
	1–2 times/week	8 (25.00)	8 (30.77)	16 (27.59)	
	3 times/week	5 (15.63)	4 (15.38)	9 (15.52)	
	4–5 times/week	4 (12.50)	1 (3.85)	5 (8.62)	
	Every day	3 (9.38)	3 (11.54)	6 (10.34)	

* *p*-value by two-sample *t*-test; ^†^ *p*-value by chi-squared test; ^‡^ *p*-value by Fisher’s exact test.

**Table 3:** Changes in uric acid in serum by visit (PP set).

		DKB114		Placebo		*p*-Value	*p*-Value ^$^
		N = 32		N = 26			
		n	Mean ± SD	n	Mean ± SD		
Uric acid in serum (mg/dL)	Baseline (visit 1)	32	7.94 ± 0.51	26	7.70 ± 0.64	0.0740 ^&^	
	6 weeks (visit 4)	32	7.47 ± 1.03	26	7.54 ± 1.01		
	Change from baseline	32	−0.47 ± 0.94	26	−0.15 ± 0.91	0.1734 ^&^	0.2966
	*p*-value **		0.0082		0.3978		
	12 weeks (visit 5)	32	7.37 ± 0.92	26	7.67 ± 0.89		
	Change from baseline	32	−0.58 ± 0.86	26	−0.02 ± 0.93	0.0229 *	0.0646
	*p*-value **		0.0007		0.9003		

Compared between groups, *p*-value by two-sample *t*-test; ^&^ Compared between groups: *p*-value by Wilcoxon rank-sum test; ** Compared within groups: *p*-value by paired *t*-test; ^$^ Compared between groups: *p*-value by ANCOVA adjusted for baseline.

**Table 4:** Proportion of subjects with uric acid concentration in serum <7.0 mg/dL (PP set).

Percentage of Subjects with Serum Uric Acid Concentration <7.0 mg/dL		DKB114	Placebo	*p*-Value †
		N = 32, n (%)	N = 26, n (%)	
Baseline (visit 1)	Less than 7.0 mg/dL	0 (0.00)	0 (0.00)	-
	More than 7.0 mg/dL	32 (100.00)	26 (100.00)	
6 weeks (visit 4)	Less than 7.0 mg/dL	12 (37.50)	5 (19.23)	0.1285
	More than 7.0 mg/dL	20 (62.50)	21 (80.77)	
12 weeks (visit 5)	Less than 7.0 mg/dL	11 (34.38)	4 (15.38)	0.1005
	More than 7.0 mg/dL	21 (65.63)	22 (84.62)	

† *p*-value by chi-squared test.

**Table 5:** Distribution of serum uric acid levels.

Uric Acid in Serum (mg/dL)	Subjects (n)			
	Visit 0		Visit 5	
	DKB114	Placebo	DKB114	Placebo
<7.0	0	0	11	4
7.0–7.5	7	13	10	6
7.6–8.0	12	4	3	8
8.1–8.5	9	4	6	5
8.6–9.0	4	5	0	2
9.0<	0	0	2	1

**Table 6:** Changes in serum xanthine oxidase activity, blood sugar, CRP (C-reactive protein), homocysteine, IL-6, NO, and apoprotein B during the study (PP set).

		DKB114		Placebo		*p*-Value ^&^	*p*-Value ^$^
		N = 32		N = 26			
		n	Mean ± SD	n	Mean ± SD		
Xanthine oxidase activity in serum (%)	Baseline (visit 2)	32	0.70 ± 0.40	26	0.84 ± 0.85	0.7486	
	6 weeks (visit 4)	32	0.74 ± 0.57	26	0.71 ± 0.93		
	Change from baseline	32	0.04 ± 0.66	26	−0.13 ± 1.35	0.4343	0.9274
	*p*-value **		0.7615		0.6233		
	12 weeks (visit 5)	32	0.74 ± 0.74	26	0.76 ± 0.48		
	Change from baseline	32	0.04 ± 0.71	26	−0.07 ± 1.02	0.8696	0.9418
	*p*-value **		0.7768		0.7109		
Blood glucose (mg/dL)	Baseline (visit 2)	32	99.34 ± 18.11	26	95.62 ± 12.87	0.6897	
	6 weeks (visit 4)	32	96.94 ± 13.16	26	96.81 ± 10.53		
	Change from baseline	32	−2.41 ± 16.34	26	1.19 ± 13.41	0.3320	0.6971
	*p*-value **		0.4111		0.6542		
	12 weeks (visit 5)	32	96.41 ± 15.92	26	94.96 ± 8.34		
	Change from baseline	32	−2.94 ± 15.32	26	−0.65 ± 11.10	0.4113	0.9112
	*p*-value **		0.2863		0.7664		
CRP (mg/dL)	Baseline (visit 2)	32	0.12 ± 0.12	26	0.14 ± 0.15	0.7279	
	6 weeks (visit 4)	32	0.12 ± 0.13	26	0.31 ± 0.75		
	Change from baseline	32	−0.01 ± 0.15	26	0.17 ± 0.73	0.2495	0.1746
	*p*-value **		0.7613		0.2402		
	12 weeks (visit 5)	32	0.17 ± 0.29	26	0.23 ± 0.31		
	Change from baseline	32	0.05 ± 0.27	26	0.10 ± 0.21	0.0443 ^&^	0.5137
	*p*-value **		0.3187		0.0324		
Homocysteine (μmol/L)	Baseline (visit 2)	32	13.69 ± 8.19	26	10.78 ± 3.47	0.0990	
	6 weeks (visit 4)	32	13.21 ± 8.21	26	10.74 ± 2.32		
	Change from baseline	32	−0.48 ± 2.36	26	−0.05 ± 2.91	0.8819	0.8788
	*p*-value **		0.2563		0.9366		
	12 weeks (visit 5)	32	12.49 ± 5.46	26	10.95 ± 2.72		
	Change from baseline	32	−1.20 ± 3.76	26	0.16 ± 2.82	0.2841	0.7375
	*p*-value **		0.0808		0.7723		
TNF-α (pg/mL)	Baseline (visit 2)	32	1.00 ± 0.32	26	0.85 ± 0.31	0.0724	
	6 weeks (visit 4)	32	0.98 ± 0.35	26	0.95 ± 0.33		
	Change from baseline	32	−0.02 ± 0.27	26	0.11 ± 0.35	0.1229	0.3942
	*p*-value **		0.6536		0.1337		
	12 weeks (visit 5)	32	0.95 ± 0.35	26	0.97 ± 0.39		
	Change from baseline	32	−0.05 ± 0.33	26	0.13 ± 0.48	0.1009	0.4023
	*p*-value **		0.3974		0.1853		
IL-6 (pg/mL)	Baseline (visit 2)	32	1.75 ± 1.07	26	1.43 ± 0.82	0.1571	
	6 weeks (visit 4)	32	1.72 ± 1.27	26	1.49 ± 0.82		
	Change from baseline	32	−0.04 ± 1.10	26	0.06 ± 0.65	0.1525	0.9909
	*p*-value **		0.8472		0.6140		
	12 weeks (visit 5)	32	1.66 ± 1.38	26	1.75 ± 1.68		
	Change from baseline	32	−0.10 ± 1.12	26	0.33 ± 1.78	0.5368	0.4342
	*p*-value **		0.6256		0.3571		
NO (μmol/L)	Baseline (visit 2)	32	56.82 ± 45.34	26	49.22 ± 33.32	0.8573	
	6 weeks (visit 4)	32	49.14 ± 34.38	26	62.04 ± 52.91		
	Change from baseline	32	−7.68 ± 49.20	26	12.82 ± 49.43	0.2082	0.1683
	*p*-value **		0.3841		0.1979		
	12 weeks (visit 5)	32	69.44 ± 53.84	26	62.11 ± 42.51		
	Change from baseline	32	12.62 ± 62.65	26	12.89 ± 44.19	0.6901	0.6956
	*p*-value **		0.2631		0.1494		
Apoprotein B (mg/dL)	Baseline (visit 2)	32	108.29 ± 23.01	26	113.73 ± 32.65	0.4611 *	
	6 weeks (visit 4)	32	104.66 ± 17.31	26	112.41 ± 33.07		
	Change from baseline	32	−3.64 ± 19.55	26	−1.32 ± 9.64	1.000&	0.3540
	*p*-value **		0.3007		0.4917		
	12 weeks (visit5)	32	103.54 ± 21.42	26	116.85 ± 30.82		
	Change from baseline	32	−4.75 ± 16.69	26	3.13 ± 12.64	0.0518 *	0.0189 ^$^
	*p*-value **		0.1175		0.2187		

* Compared between groups: *p*-value by two-sample *t*-test; ^&^ Compared between groups: *p*-value by Wilcoxon rank-sum test; ** Compared within groups: *p*-value by paired *t*-test; ^$^ Compared between groups; *p*-value for ANCOVA adjusted for baseline.

**Table 7:** Serum chemistry tests (safety set).

		DKB114		Placebo		*p*-Value *
		N = 32		N = 26		
		n	Mean ± SD	n	Mean ± SD	
Total cholesterol (mg/dL)	Baseline (visit 2)	40	201.38 ± 32.93	40	202.80 ± 48.16	0.8777
	12 weeks (visit 5)	36	194.03 ± 30.35	36	213.22 ± 47.11	
	Change from baseline	36	−5.94 ± 17.00	36	10.03 ± 19.72	0.0005 *
	*p*-value **		0.0432		0.0043	
LDL-cholesterol (mg/dL)	Baseline (visit 2)	40	121.05 ± 28.52	40	122.80 ± 40.30	0.8235
	12 weeks (visit 5)	36	112.17 ± 25.15	36	129.47 ± 39.21	
	Change from baseline	36	−8.89 ± 19.10	36	6.31 ± 21.07	0.0020 *
	*p*-value **		0.0084		0.0812	
HDL-cholesterol (mg/dL)	Baseline (visit 2)	40	54.26 ± 14.49	40	47.63 ± 10.87	0.0232
	12 weeks (visit 5)	36	52.68 ± 11.55	36	48.87 ± 10.45	
	Change from baseline	36	−0.39 ± 7.23	36	0.82 ± 6.35	0.4548
	*p*-value **		0.7506		0.4439	
Triglycerides (mg/dL)	Baseline (visit 2)	40	178.78 ± 139.42	40	243.33 ± 256.06	0.1666
	12 weeks (visit 5)	36	183.11 ± 139.99	36	226.42 ± 152.07	
	Change from baseline	36	11.44 ± 77.71	36	−7.36 ± 245.47	0.6635
	*p*-value **		0.3829		0.8582	
HbA1c (%)	Baseline (visit 2)	40	5.42 ± 0.33	40	5.49 ± 0.50	0.4437
	12 weeks (visit 5)	36	5.55 ± 0.32	36	5.48 ± 0.30	
	Change from baseline	36	0.12 ± 0.16	36	0.08 ± 0.14	0.3405
	*p*-value **		0.00001		0.0009	
AST(GOT) (IU/L)	Baseline (visit 2)	40	27.78 ± 139.42	40	26.13 ± 6.71	0.1666
	12 weeks (visit 5)	36	29.14 ± 14.67	36	33.78 ± 42.16	
	Change from baseline	36	0.83 ± 13.06	36	7.78 ± 41.84	0.3473
	*p*-value **		0.7041		0.2723	
ALT(GPT) (IU/L)	Baseline (visit 2)	40	33.15 ± 15.06	40	29.93 ± 11.61	0.2867
	12 weeks (visit 5)	36	36.64 ± 31.00	36	34.06 ± 23.75	
	Change from baseline	36	3.44 ± 22.29	36	3.67 ± 20.20	0.9648
	*p*-value **		0.3602		0.2835	
BUN (mg/dL)	Baseline (visit 2)	40	13.84 ± 3.46	40	13.44 ± 3.27	0.5964
	12 weeks (visit 5)	36	14.38 ± 4.01	36	14.14 ± 3.87	
	Change from baseline	36	0.62 ± 3.68	36	0.94 ± 3.79	0.7202
	*p*-value **		0.3192		0.1477	
Creatinine (mg/dL)	Baseline (visit 2)	40	1.02 ± 0.16	40	1.02 ± 0.14	0.8028
	12 weeks (visit 5)	36	0.98 ± 0.14	36	1.02 ± 0.14	
	Change from baseline	36	−0.04 ± 0.10	36	0.00 ± 0.08	0.0700
	*p*-value **		0.0374		0.8631	

* Compared between groups: *p*-value by two-sample *t*-test; ** Compared within groups: *p*-value by paired *t*-test.

**Table 8:** Vital signs (heart rate, blood pressure) and weight change (safety set).

		DKB114		Placebo		*p*-Value *
		N = 32		N = 26		
		n	Mean ± SD	n	Mean ± SD	
Heart rate (count/min)	Baseline (visit 3)	40	75.33 ± 9.45	40	77.15 ± 9.56	0.3932
	12 weeks (visit 5)	36	77.69 ± 11.62	36	78.69 ± 13.02	
	Change from baseline	36	2.58 ± 7.54	36	1.14 ± 11.96	0.5422
	*p*-value **		0.0474		0.5713	
Systolic pressure (mmHg)	Baseline (visit 3)	40	128.80 ± 11.82	40	125.58 ± 10.38	0.1987
	12 weeks (visit 5)	36	128.22 ± 10.21	36	127.06 ± 10.67	
	Change from baseline	36	0.14 ± 11.57	36	2.14 ± 10.49	0.4448
	*p*-value **		0.9430		0.2292	
Diastolic pressure (mmHg)	Baseline (visit 3)	40	80.40 ± 10.17	40	78.90 ± 7.97	0.4650
	12 weeks (visit 5)	36	79.47 ± 8.72	36	81.31 ± 8.14	
	Change from baseline	36	0.47 ± 6.98	36	2.39 ± 10.12	0.3532
	*p*-value **		0.6873		0.1655	
Weight (kg)	Baseline (visit 3)	40	82.56 ± 15.27	40	79.24 ± 12.47	0.2904
	12 weeks (visit 5)	36	80.91 ± 12.92	36	80.21 ± 13.18	
	Change from baseline	36	−0.38 ± 3.98	36	0.52 ± 2.38	0.2518
	*p*-value **		0.5724		0.2016	

* Compared between groups: *p*-value by two-sample *t*-test; ** compared within groups: *p*-value by paired *t*-test.
